# *Wolbachia* and Lymphatic Filarial Nematodes and Their Implications in the Pathogenesis of the Disease

**DOI:** 10.1155/2024/3476951

**Published:** 2024-05-02

**Authors:** Abebaw Setegn, Gashaw Azanaw Amare, Yenesew Mihret

**Affiliations:** ^1^Department of Medical Parasitology, University of Gondar, Gondar, Ethiopia; ^2^Department of Medical Laboratory Sciences, Debre Markos University, Debre Markos, Ethiopia

## Abstract

Lymphatic filariasis (LF) is an infection of three closely related filarial worms such as *Wuchereria bancrofti*, *Brugia malayi*, and *Brugia timori*. These worms can cause a devastating disease that involves acute and chronic lymphoedema of the extremities, which can cause elephantiasis in both sexes and hydroceles in males. These important public health nematodes were found to have a mutualistic relationship with intracellular bacteria of the genus *Wolbachia*, which is essential for the development and survival of the nematode. The host's inflammatory response to parasites and possibly also to the *Wolbachia* endosymbiont is the cause of lymphatic damage and disease pathogenesis. This review tried to describe and highlight the mutualistic associations between *Wolbachia* and lymphatic filarial nematodes and the role of bacteria in the pathogenesis of lymphatic filariasis. Articles for this review were searched from PubMed, Google Scholar, and other databases. Article searching was not restricted by publication year; however, only English version full-text articles were included.

## 1. Introduction

Lymphatic filariasis is a neglected tropical disease caused by the filarial nematodes of *Wuchereria bancrofti*, *Brugia malayi*, and *Brugia timori* [1, 2], which are transmitted through the bite of mosquitoes of the genus *Anopheles*, *Aedes*, *Culex*, and *Mansonia* [2].

Lymphatic filariasis is the second main parasitic cause of disability with an estimated 5.549 million disability-adjusted life years [3], and about 51 million people are affected worldwide [4]. Filariasis caused by *W. bancrofti* accounts for approximately 90% of cases of lymphatic filariasis, and the remaining 10% is caused by the two *Brugia* species [5]. The clinical manifestation of the disease can range from severe swelling, usually in the limbs (lymphedema) or scrotal sac (hydrocele), to occurrences of acute adenolymphangitis [1]. These complications of lymphatic filariasis cause substantial morbidity in the world [6]. Additionally, this illness increases susceptibility to opportunistic infections [7], especially when lymphedema from a persistent filarial infection advances. Opportunistic microorganisms that cause long-term recurrent secondary infections are what lead to the development of elephantiasis [8].

Community-level transmission of lymphatic filariasis can be limited by mass therapy with prescribed oral regimens of antihelminthic drugs such as albendazole, alone or with ivermectin, or diethylcarbamazine citrate and albendazole, or a combination of all three [9, 10].

*Wolbachia* are obligate endosymbiotic *α*-proteobacteria that are polymorphic and closely related to other rickettsial organisms such as *Rickettsia*, *Ehrlichia*, and *Anaplasma* [11, 12]. Current evidence in filarial research reveals that endosymbiotic *Wolbachia* bacteria play a significant role in the biology of filarial nematodes [13]. The symbiotic associations of *Wolbachia* and its role in the pathogenesis of lymphatic filariasis are discussed below.

## 2. Mutualistic Association between *Wolbachia* and Lymphatic Filarial Nematodes

An intimate type of symbiotic interaction known as endosymbiosis occurs when one organism lives inside the body of another, establishing a range of relationships from parasitism to compulsory mutualism [14].

*Wolbachia* is a common and abundant endosymbiotic bacteria that lives in the vacuoles of host germline and somatic cells and is found in arthropods and filarial nematodes [15].

*Wolbachia* spreads vertically from the mother to the offspring in both nematodes and arthropods. These bacteria are present in oocytes before fertilization; during host development, differences in the tropism of certain *Wolbachia* are noted; nevertheless, the female reproductive system is a common site in all animal hosts [16]. In filarial nematodes, somatic tissues, such as the lateral cords, are also heavily colonized in addition to their placement in the ovary, which is crucial for maternal transmission [17].

It has been demonstrated that *Wolbachia* is present in the majority of filarial worms that cause serious sickness in both humans and animals, including *W. bancrofti*, *B. malayi*, *Onchocerca volvulus*, *Dirofilaria immitis*, and *Dirofilaria repens* [17].

*Wolbachia* has the ability to significantly alter hosts' biology. It can identify changes in host reproduction in arthropods, such as parthenogenesis, feminization of genetic males, male embryo death, and cytoplasmic incompatibility (CI) [16]. Furthermore, there are unusual *Wolbachia*-insect partnerships in which the bacteria is necessary for host reproduction. Thus, there is evidence that *Wolbachia* is not only involved in the changing of host reproduction, but it also seems to be necessary for the host arthropod in certain circumstances. There exist instances of bacteria-insect relationships where the bacteria have a role in controlling iron homeostasis [18], or in other circumstances, these symbionts provide defense against viral infections [19].

It is most likely connected to the induction of insect immunity that *Wolbachia* offers as a defense mechanism against insect viruses. Recent research has demonstrated that infecting *Aedes aegypti* with the *Wolbachia* popcorn strain strongly activates the mosquito's immunity, which in turn protects the insect against infections by parasites including malaria and filarial worms [20, 21]. *Wolbachia* bacteria have also been found in filarial nematodes of animals, including filariae infecting cattle (*Onchocerca gutturosa* and *Onchocerca lienalis*) [22, 23].

The bacteria is found to be an obligatory symbiont in filarial worms and is needed for nematode development, reproduction, and long-term survival. This is supported by pioneer studies, which stated that treatments with antibiotics directed against the bacteria (tetracycline and its derivatives) had a number of harmful effects on the host nematode; the most notable effects were inhibition of embryogenesis and the production of microfilaria, as well as inhibition of development from infectious larvae (L3) to adults. Adulticide effects were also found in one of these groundbreaking research, including the cow filarial *Onchocerca ochengi* [22].

*Wolbachia* exhibit a wide range of symbiotic relationships with their hosts, from commensal, parasitic, or harmful relationships with insects and other arthropod hosts to obligate mutualism in filarial nematodes [15].

The deoxyribonucleic acid (DNA) sequencing data revealed that *W. bancrofti* and *B. malayi*, which are the primary species for lymphatic filariasis, were found to include an internal bacterium that resembled *Wolbachia* [24]. Furthermore, these lymphatic filarial nematodes were found to be infected with *Wolbachia* [25]. All stages of the life cycle of filarial worms are infected with this bacteria, but the severity of infections differs between different stages of the nematodes [26, 27].

This obligate mutualistic symbiosis relationship between *Wolbachia* and filarial worms [28] is based on metabolic complementarity and increases or strengthens one or both host's biochemical variety and pathways [29, 30]. For instance, *Wolbachia* is necessary for healthy larval growth and development, embryogenesis, and adult worm survival [17], while the nematode host provides the amino acids required for the bacteria's growth [28].

Data from the sequencing of the genomes of both *Wolbachia* (wBm) and its nematode host *B. malayi* revealed that for a wide variety of biological processes, including the synthesis of metabolites such as haem, riboflavin, flavin adenine dinucleotide, and nucleotides, *Wolbachia* provides to nematodes because they cannot synthesize these molecules on their own [28].

In addition, there are biological processes such as the growth and development of larvae and adult female embryogenesis; the nematode's rapid growth, development, and organogenesis; and its association with the rapid expansion of *Wolbachia* populations following larval infection of mammalian hosts and in reproductively active adult females which are impossible in the absence of *Wolbachia* because all these activities have a high metabolic demand [26].

On the other hand, due to the absence of *Wolbachia*, extensive apoptosis occurs in the germline and somatic cells of embryos, microfilariae, and fourth-stage larvae. This is most likely because these cells and tissues do not have critical nutrients or metabolites that would prevent apoptosis [31].

Furthermore, a comparison of genomes also indicates that *Wolbachia* shows a metabolic dependence on the nematode host in numerous vitamins and cofactors for its growth, including coenzyme A, nicotinamide adenine dinucleotide, biotin, ubiquinone, folate, lipoic acid, and pyridoxal phosphate, which the bacteria did not synthesize de novo [28]. *Wolbachia* also has importance for the filarial nematodes in which the bacteria has an enzyme called *Wolbachia* catalase, which might protect both the host of the nematode and the bacteria from oxidative damage [32].

In general, since *Wolbachia* spread vertically through oocytes in filarial nematodes, the internal endosymbiont will be absent after worm sterilization. Therefore, the viability of the filarial worms will be affected without *Wolbachia*. This makes the bacteria a fascinating target for filarial medication treatment due to all of these characteristics [31]. This is supported by a number of studies in both animal and *in vitro* settings, which showed that *Wolbachia* are a promising therapeutic target for human filariasis [17].

Early preclinical researches revealed that adult filarial worms may die, experience developmental retardation, and experience embryotoxicity as a result of antibiotic therapy directed against the endosymbiont bacteria [33]. The adult germline and somatic cells in the embryos and microfilariae undergo substantial apoptosis as a result of the subsequent depletion of *Wolbachia*, which sterilizes the filarial nematodes [31]. As a result, it seemed that *Wolbachia* was the ideal target for treating human filarial diseases [34].

## 3. The Implications of *Wolbachia* in the Pathogenesis of Lymphatic Filariasis

Lymphatic filarial worms can cause a variety of infection manifestations related to serious and long-lasting inflammation [35]. The main source of knowledge on the role of *Wolbachia* in the pathogenesis of human lymphatic filarial infections is research on the molecular pathogenesis of inflammation caused by filarial worms [36].

The stimulation of proinflammatory and immunomodulatory processes in the host is one way that *Wolbachia* is involved in the infection process, from the acute phase to the development of chronic problems [36]. A defense mechanism against molecular structures that are shared by many different types of organisms is the innate immune system. It involves identifying certain “markers,” or pathogen-associated molecular patterns (PAMPs), that indicate the existence of “generic” pathogens [37].

Subsequently, Toll-like receptors (TLRs) on the surface of antigen-presenting cells recognize these PAMPs, and proinflammatory cytokines and reactive oxygen species are produced, along with an upregulation of costimulatory molecules that aid in the development of an adaptive immune response [37]. Both Th1-adaptive immune responses and innate inflammatory responses may be activated by the release of *Wolbachia* lipoprotein [38].

*Wolbachia* is discharged after the worm dies in hosts infected with filarial nematodes carrying the bacteria through a larval moult, natural attrition, microfilarial turnover, and pharmaceutical interventions. The release of the bacteria is involved in both increasing inflammatory-mediated pathogenesis and the hyporesponsiveness of the immune system [17].

The bacteria can cause an inflammatory response by interacting with immune cells such as neutrophils, dendritic cells, and monocytes or macrophages [39]. This role can be simulated *in vitro* by exposing innate immune cells to parasite extracts. Antigen-presenting cells challenged with complete *Wolbachia*-containing worm extracts produce significant amounts of inflammatory cytokines, while stimulation with *Wolbachia* antibiotic-depleted worm extracts or with *Wolbachia*-free filariae extracts does not result in such a reaction [38, 40, 41].

Additionally, a number of molecular pattern possibilities associated with pathogens for *Wolbachia* have been shown to modulate the inflammatory response, such as diacylated lipoproteins, which promote Th1 polarization and the innate inflammatory response [38]. The bacteria also prevents eosinophils from degranulating, which is necessary for the eradication of filarial infections. This affects the neutrophil response of the host. By providing immunity against the fatal effector cell response, *Wolbachia*'s modulation of the local inflammatory response prolongs the life of the nematode host through defensive mutualism [42].

Patients with human lymphatic filariasis with *B. malayi* experience severe systemic inflammatory reactions that are strongly correlated with the discharge of bacteria into their blood after antifilarial therapy of worms [43]. This gives concrete proof that upon the death of the nematode, *Wolbachia* is released into the blood and exposed to the host's immune systems. Studies in *B. malayi-*infected animals have revealed a further connection between immunological reactions to bacteria and the emergence of lymphatic filarial diseases [43].

A *Wolbachia* surface protein (WSP) and the antibody response to the protein are correlated with the onset and duration of episodes of inflammatory lymphedema [44]. This result supports the hypothesis that innate and acquired immune responses to endosymbiont bacteria could be involved in the pathogenesis of lymphatic filarial disease because endosymbiont antigens are recognized by the acquired immune response [45].

By activation of Toll-like receptors-2 (TLR-2) with extracts of *B. malayi*, lymphangiogenic factors such as angiopoietin-1 and vascular endothelial growth factor are also produced [46]. The severity of the infection was found to be correlated with the increased levels of these lymphangiogenic agents [46, 47]. The innate immune receptor TLR-4 identifies and binds to lipopolysaccharide (LPS), an antigenic element of gram-negative bacteria's outer leaflet [48].

Following ligand attachment, TLR-4 triggers a signaling cascade that activates critical intracellular transcription factors such as interferon response factor (IRF), activator protein-1 (AP-1), and nuclear factor kappa B (NF-*κ*B) [49]. It has been discovered that the filarial nematode's bestrophin homolog and *Wolbachia* endosymbiont's LPS serve as ligands for TLR-4, causing inflammation in the macrophage population and ultimately causing filarial lymphedema [50].

Recent reports revealed the existence of a novel Toll-like receptor-4 (TLR-4) ligand called microfilarial protein (MfP) from the sheath of microfilariae (the larval form) of the human filarial worm *W. bancrofti*. The microfilarial protein activates the TLR-4-NF-*κ*B pathway in macrophages, causing a proinflammatory response [50]. Antagonists of lipid A are found to prevent pattern recognition receptors CD14 and TLR-4 from activating innate inflammatory responses [39]. When worms are treated with an antifilarial medication in human filariasis, the release of bacteria into the blood is highly linked to severe systemic inflammatory reactions in patients with *B. malayi* infection [43].

Proinflammatory cytokines and inflammatory mediators, such as interleukin-1b (IL-1b), IL-6, interferon-*ϒ* (IFN-*ϒ*), tumor necrotic factor-*α* (TNF-*α*), nitric oxide (NO), and LPS binding protein (LBP), are released in conjunction with adverse drug reactions, which are more common in those individuals with high microfilarial burdens [51–53]. This implies that the discharge of bacteria as a result of parasite death stimulates inflammatory responses, leading to acute inflammatory conditions related to the death of adult worms and adversative reactions to drugs [45].

The existence of significant concentrations of inflammatory cytokines, like IL-1b, IL-6, IL-8, TNF-a, and granulocyte-macrophage colony-stimulating factors in fluids from limb lymphoedema and hydroceles, provides evidence for the involvement of inflammatory responses in the development of chronic disease [54].

Anti-inflammatory mediators such as IL-4, IL-10, IL-13, transforming growth factor- (TGF-) *β*, IL-1RA, glucocorticoids, prostaglandin E-2, and proinflammatory cytokine soluble receptors control the generation of proinflammatory responses after exposure to lipopolysaccharide (LPS) [55]. Although this is supposed to shield against the uncontrolled immune activation of acute endotoxic shock, it can lead to an inability to respond appropriately to secondary infections in survivors of endotoxic shock [45].

**Figure 1:**
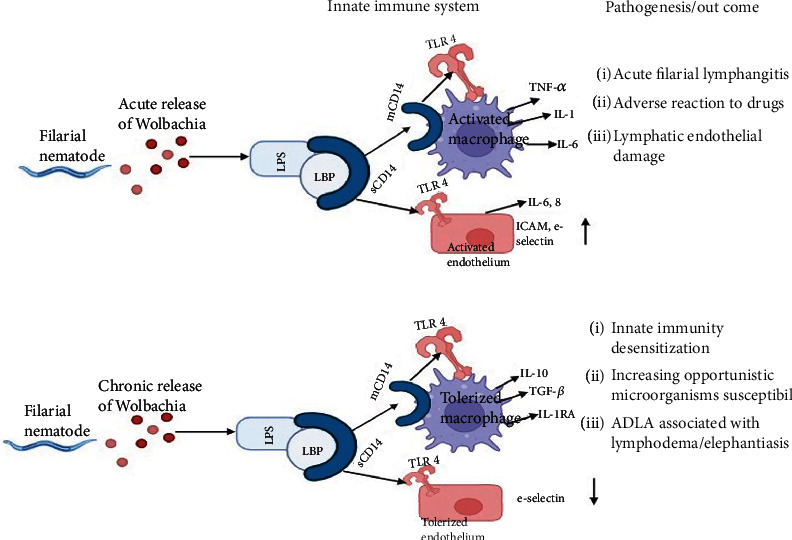
Schematic representation of *Wolbachia* bacteria in the development of the immunopathology of lymphatic filarial diseases (adapted from [45]).

Moreover, the innate immune system cells may become desensitized because of the chronic release of *Wolbachia*. This chronic release, besides the harm caused by acute inflammatory episodes to the structure and functionality of parasitized lymphatics, would reassure the emergence of environmental opportunistic infections such as those that arise during acute dermatolymphangioadenitis (ADLA), which in turn is linked to chronic lymphodema and elephantiasis (Figure 1) [56, 57].

Anti-*Wolbachia* doxycycline treatment has led to a reduction in scrotal lymphatic vessel enlargement in patients with *W. Bancrofti* infection, which was indicative of an improvement in symptoms of the disease [58]. This provides evidence in favor of using *Wolbachia* as a therapeutic target for the treatment of filarial pathology that doxycycline may have a healing effect in patients with filarial pathology [59].

Doxycycline also has effects on filarial worms, independent of *Wolbachia* bacteria. This is supported by in vivo studies on *the* effects of doxycycline on gene expression in *Wolbachia* and *Brugia malayi* adult female worms which state that the drug has effects on the downregulation of *B. malayi* genes which are involved in the development and reproduction of embryos. These genes are necessary for growth, development, and reproduction [60].

However, following doxycycline therapy, there were enhanced expression signals for numerous *B. malayi* genes related to energy synthesis, electron transport, metabolism, antioxidants, and other unknown functions. These findings imply that, although without the ability to reproduce, female worms are able to compensate for the loss of *Wolbachia* in order to survive [60].

Furthermore, an extremely efficient way to keep an eye on human filariasis infections would be to target *Wolbachia*. In 2009, the first computational attempt was made to pinpoint the vital genes of the endosymbiotic bacterium *Wolbachia*, which is not cultivable [61].

Currently, the whole genome of *Wolbachia* from *Brugia malayi* (wBm) is already available, which may be used to determine the best treatment targets in wBm using a hierarchical proteome subtractive method [28]. Some of the potential drug targets of *Wolbachia* endosymbiont (*Brugia malayi*) are outer membrane protein, N5-carboxyaminoimidazole ribonucleotide synthase, and UDP-N-acetylenolpyruvoylglucosamine reductase [62].

## 4. Conclusions

As we have discussed in our review, we found that *Wolbachia* bacteria play a necessary role in the biology of lymphatic filarial nematodes. The symbiotic association between the bacteria and filarial nematodes is crucial for biological processes, such as transmission through the vector and the longevity of adult worms, in addition to the high metabolic demand during growth and development. Several studies on the inflammation-based lymphatic filariasis pathogenesis have revealed that the main inflammatory provocation for lymphatic filarial worms is endotoxin-like action obtained from endosymbiont bacteria, and *Wolbachia* bacterial genes become the main drug targets in human filariasis infections.

## Data Availability

All relevant data are fully available without restriction within the manuscript.
